# Gum Tragacanth (GT): A Versatile Biocompatible Material beyond Borders

**DOI:** 10.3390/molecules26061510

**Published:** 2021-03-10

**Authors:** Mohammad Ehsan Taghavizadeh Yazdi, Simin Nazarnezhad, Seyed Hadi Mousavi, Mohammad Sadegh Amiri, Majid Darroudi, Francesco Baino, Saeid Kargozar

**Affiliations:** 1 Medical Toxicology Research Center, School of Medicine, Mashhad University of Medical Sciences, Mashhad 917794-8564, Iran; ehsan3753@yahoo.com (M.E.T.Y.); Mousavih@mums.ac.ir (S.H.M.); 2 Tissue Engineering Research Group (TERG), Department of Anatomy and Cell Biology, School of Medicine, Mashhad University of Medical Sciences, Mashhad 917794-8564, Iran; smn.nazarnezhad@yahoo.com; 3 Department of Biology, Payame Noor University, Tehran 43183-1455, Iran; amiriherb@gmail.com; 4 Nuclear Medicine Research Center, Mashhad University of Medical Sciences, Mashhad 917794-8564, Iran; Darroudim@mums.ac.ir; 5 Applied Science and Technology Department, Institute of Materials Physics and Engineering, Politecnico di Torino, Corso Duca degli Abruzzi 24, 10129 Torino, Italy

**Keywords:** gum tragacanth, biomaterials, natural polymers, green chemistry, biomedical engineering, tissue engineering, wound healing

## Abstract

The use of naturally occurring materials in biomedicine has been increasingly attracting the researchers’ interest and, in this regard, gum tragacanth (GT) is recently showing great promise as a therapeutic substance in tissue engineering and regenerative medicine. As a polysaccharide, GT can be easily extracted from the stems and branches of various species of *Astragalus.* This anionic polymer is known to be a biodegradable, non-allergenic, non-toxic, and non-carcinogenic material. The stability against microbial, heat and acid degradation has made GT an attractive material not only in industrial settings (e.g., food packaging) but also in biomedical approaches (e.g., drug delivery). Over time, GT has been shown to be a useful reagent in the formation and stabilization of metal nanoparticles in the context of green chemistry. With the advent of tissue engineering, GT has also been utilized for the fabrication of three-dimensional (3D) scaffolds applied for both hard and soft tissue healing strategies. However, more research is needed for defining GT applicability in the future of biomedical engineering. On this object, the present review aims to provide a state-of-the-art overview of GT in biomedicine and tries to open new horizons in the field based on its inherent characteristics.

## 1. Introduction

Gums are known to be pathological products generated after plant injuries or due to unfavorable conditions (e.g., drought) through the breakdown of cell walls (extracellular formation; gummosis). Polysaccharide gums are ones of the most abundant raw materials in nature. Besides being renewable sources, they are easily accessible, relatively affordable, non-toxic, and environmentally friendly, causing their worldwide usage from the food industry to health care systems. Among different well-characterized gums, gum tragacanth (GT) is recognized as a versatile material in biomedicine. Generally, GT, also known as Katira, is sourced from Central Asia and Eastern countries, and Iran is the largest producer and exporter of this natural gum [1,2].

Structurally, there are two general types of GT, ribbon (the best grades) and flake (or harmony). After collection, Iranian tragacanth ribbons are sorted into five grades, while flakes are provided in seven different grades [3,4]. Based on the literature, physico-chemical properties and compositional variations of GT depend on its sources, i.e., different types of *Astragalus* species [5]. *Astragalus gummifer* has been previously the primary source of GT, while *Astragalus microcephalus* is currently considered as the major source [6]. Other valuable sources to supply GT are *Astragalus gummifer* Labill., *Astragalus verus* Olivier, *Astragalus microcephalus* Willd., *Astragalus brachycalyx* Fisch. ex Boiss., *Astragalus myriacanthus* Boiss., *Astragalus echidna* Bunge and *Astragalus kurdicus* Boiss [7].

GT has been found a useful plant-derived molecule in a wide range of healthcare-related applications, such as lotions applied for external applications (hair and hand creams) [8,9]. Due to its remarkable stability in wide ranges of pH and temperatures, GT is commonly used as an emulsifier in food, drugs and related industries with exceptionally long shelf life [10]. For instance, GT is being applied as an emulsifying/suspending agent in pharmacological industries. Moreover, GT has been historically used as an analgesic as well as a conventional therapy in the curing of cough and lip fissures [8]. In modern medicine concepts, GT could be utilized for preparing tissue-engineered (TE) constructs (scaffolds) as well as in the fabrication of drug delivery platforms [11] thanks to its excellent inherent features, including non-mutagenicity, non-teratogenicity, non-immunogenicity, and non-toxicity [12]. Accordingly, GT has been generally recognized as a safe (GRAS) substance by the Food and Drug Administration (FDA). Degradability in the living systems also makes GT a highly interesting material in tissue engineering and regenerative medicine strategies [13]. Therefore, several experimental studies can take benefit from GT for fabricating wound dressings [14,15]. In addition to soft tissue healing applications, GT has been using in the reconstruction of hard tissues either alone or embedded within composites [16,17]. For instance, Haeri et al. in 2017 reported that GT could serve as a suitable substrate for promoting the adhesion, proliferation, and osteogenic differentiation of adipose-derived mesenchymal stem cells (Ad-MSCs) [16].

**Figure 1:**
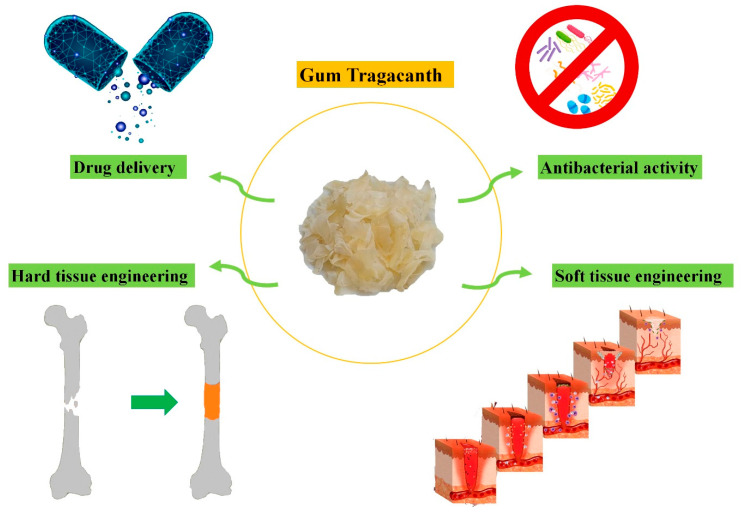
GT offers excellent opportunities for biomedical applications regarding its appropriate physicochemical and biological properties. This natural substance is now being applied for a broad range of applications, from drug delivery strategies to hard and soft tissue engineering.

In the present review, we aim to highlight the biological benefits of GT in biomedicine (see Figure 1) and critically analyze the limitations on the way of the extensive usage of this natural biomaterial in tissue engineering and regenerative medicine applications. For this purpose, physico-chemical and biological properties of GT are first summarized, and then the results of in vitro and in vivo evaluations of GT, either alone or in combination with other materials, will be discussed.

## 2. Research Methodology

The relevant information on the GT was obtained from scientific databases, including Web of Science, Scopus, and PubMed. The search was performed by using the following keywords: gum tragacanth, tissue engineering, hard tissue engineering, soft tissue engineering, bone tissue engineering, skin regeneration, and wound healing. In this study, scientific and author names of plant species are reported according to the most recent monograph of the genus [18].

## 3. Physico-Chemical Properties of GT

### 3.1. Chemical Composition and Structure of GT

**Figure 2:**
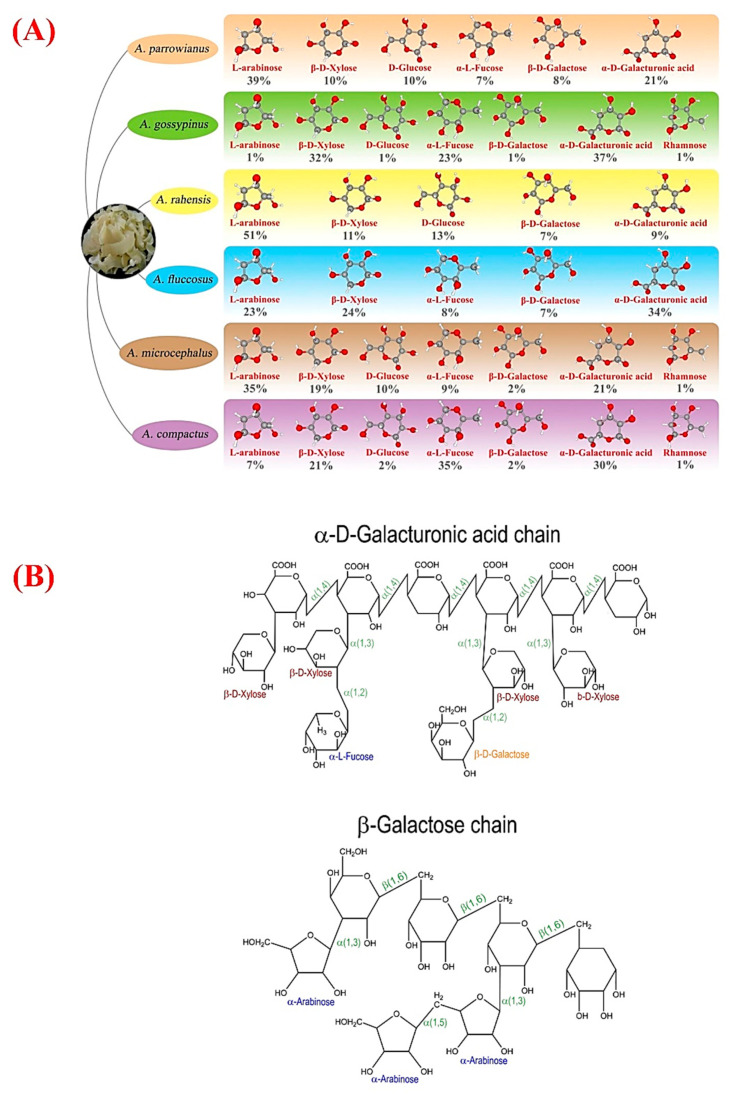
(**A**) Comparison of major chemical components of different GT species. (**B**) Proposed chemical structure of GT, including xylogalacturonan (top) and arabinogalactan (down). With permission from [23] Copyright 2020, Elsevier.

As mentioned in the Introduction, the composition of GT strongly depends on the *Astragalus* species used as a source (Figure 2A). For instance, the chemical composition of the commercial GTs attained from different species shows significant differences, which are directly resulted from seasonal and geographical variations [4]. GT has a slightly acidic nature with a molecular weight (MW) of up to 850 kDa. Experimental research showed that GT could be notably efficient as a viscosity enhancer and stabilizer in acidic solutions [19]. Its moisture content for different species is in the range of 8.79–12.94 g/100 g of product and generates highly viscous solutions when dispersed in water. The protein content also shows different values depending on the species; for example, *A. fluccosus*, *A. microcephalus*, and *A. compactus* may typically contain 1.65–2.59% protein in their composition. In addition, the carbohydrate content of different species varied in the range of 83.81–86.52%. Although there are variations in the mineral content of GT species, calcium and potassium are the main inorganic elements for all species [20].

Tragacanthin and bassorin are recognized as the two main fractions in GT, and their different ratios lead to diverse physico-chemical and rheological properties of GT [21]. In more detail, tragacanthin is composed of tragacanthic acid containing residues of D-galacturonic acid, D-xylose, L-fucose, and D-galactose and an arabinogalactan (containing residues of L-arabinose, D-galactose, and D-galacturonic acid) (see Figure 2B) [22].

By adding to water, the soluble fractions of GT (i.e., tragacanthin or tragacanthic acid) dissolve and yield the formation of a viscous colloidal hydrosol, while bassorin (60–70%) is formed as an insoluble fraction of the gel [24]. The chemical structures of these fractions have been extensively studied [25,26,27]. Under the same conditions, the viscosity of bassorin is higher than that recorded for tragacanthic acid, and the viscosity of GT is between the values of the two components. GT is considered as a pseudoplastic material that shows a non-Newtonian fluid behavior as its viscosity decreases under the shear strain (shear thinning) [19,28]. The aqueous solution of GT is the most viscous substance among the natural plant gums, with excellent heat stability [29]. The viscosity of GT differs along with changing ionic strength as well as pH and temperature values due to many carboxylic groups in its structure (ranging from 0.002 up to near 4 Pa·s); the highest viscosity recorded is between pH 5 to 6. The viscosity of GT solutions decreases with a decrease in pH due to the reduction in ionic dissociation of the carboxylic groups [30]. Investigation of the water absorption properties of different gums from 20 to 65 °C has revealed that GT has higher water absorption compared to guar gum and locust bean gum [31]. It has been well-documented that differences in hygroscopic properties of gums result from the presence of various acidic and ionic units in their structure [31]. The type of initiator, monomer, and crosslinking agent applied in the synthesis were identified as the main determinants in the water absorbance property of a GT-based superabsorbent (water-absorbent equivalent to 864 g/20 mL of water/absorbent) [32]. The gel content of a tyramine-conjugated GT hydrogel synthesized by electron beam irradiation was found to be 75–85% in another study [33,34]. GT is currently being used in different areas of science and technology, including food processing, cosmetics, and the pharmaceutical industry, thanks to its emulsifying ability, excellent thermal stability, long shelf life, as well as excellent solubility and rheological behavior [35]. This polysaccharide is long-lasting over a wide range of pH and absorbs water well due to its hydrophilicity. It is biocompatible and safe for oral intake [36,37]. GT also exhibits nephron protective properties against possible nephrotoxic substances [38]. Solution properties of the water-soluble part of GT were studied by gel permeation chromatography (GPC) combined with multi-angle light scattering and viscosimetry at 25 °C. The results obtained showed that bassorin and tragacanthin exhibited quite different rheological properties. A 1% bassorin solution at 25 °C shows a high viscosity with a gel-like structure; however, the tragacanthin solution behaves like semidilute to a concentrated solution of entangled, random coil polymers.

### 3.2. Degradation of GT

Possible degradation mechanisms for GT include enzymatic degradation as well as ultrasonic waves [39,40]. Gavlighi et al. could successfully depolymerize GT by using *A. niger* pectinases and divided it into three molecular weight (MW) fractions, including HAG1 (MW < 2 kDa); HAG2 (2 kDa < MW < 10 kDa) and HAG3 (MW > 10 kDa) [41]. The authors showed that these fractions did not exert any significant effect on viscosity and could be used as natural functional food ingredients. In 2019, Raoufi et al. applied ultrasonic treatment for the degradation of GT and evaluated its impact on chain conformation and molecular properties of GT [40]. They were able to solubilize GT without any undesirable change in the primary structure or the building repeating blocks. In addition to ultrasonic treatment, the use of gamma rays has also been effective in terms of GT degradation, with no significant alteration in its chemical structure [42]. In the concept of biomedical engineering, it is necessary to determine the exact molecular mechanisms behind the degradation of GT, especially in the human body; therefore, more research is required to address this important but still ignored issue in the field.

### 3.3. Modification of GT

Generally, the inherent properties of GT can be improved via a series of modification approaches, either physical (e.g., thermal treatments) or chemical (e.g., crosslinking by Ca^2+^ and Ba^2+^ ions) methods [43]. Having carboxylic and hydroxyl groups, GT is mentioned as a desirable substance for creating linkages with various functional groups including amino, carboxyl, hydroxyl, and sulfonic acids [44]. On this point, ionic crosslinkers and organic monomers have been applied to make the ionic linkage with the COOH groups available in the GT structure [45,46]. In addition, chemical reagents, including glycerin, ethylene glycol, triethylene glycol, and glutaraldehyde, have also been used as crosslinking agents [47]. It should be stated that other processes for GT functionalization are available, including grafting, interpenetrating network formation, and blending [33,48,49].

## 4. GT in Green Chemistry

**Figure 3:**
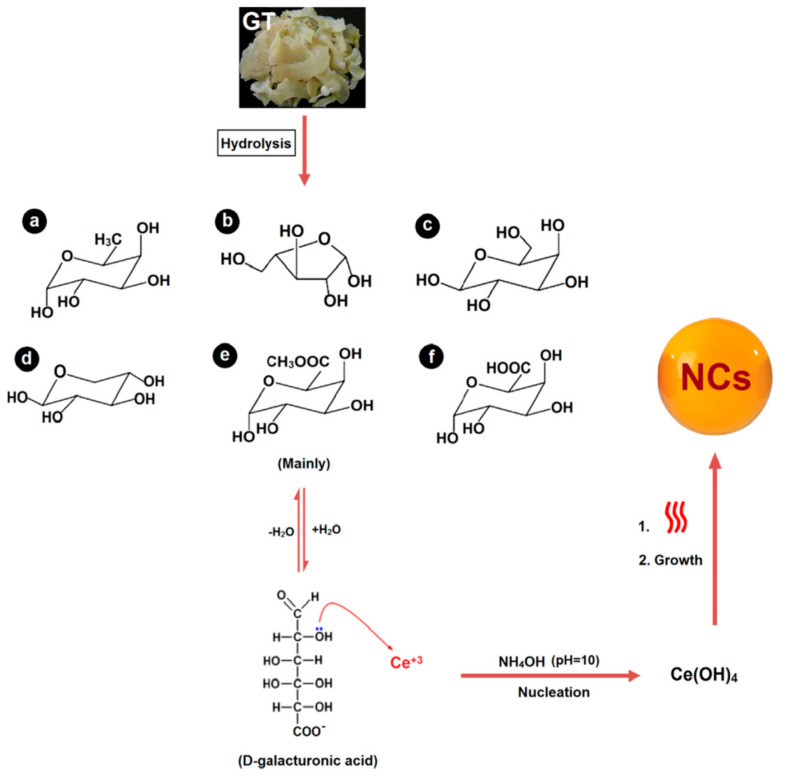
Schematic representation showing the usability of GT in the green synthesis of nanoceria particles (NCs): (**a**) α-L-fucose, (**b**) L-arabinose, (**c**) β-D-galactose, (**d**) β-D-xylose, (**e**) α-D-Galacturonic acid methyl ester, and (**f**) α-D-galacturonic. Reproduced from ref [54], Copyright 2014, Elsevier.

Green chemistry utilizes a set of “sustainable” principles with the goal of reducing or eliminating hazardous substances in the design, synthesis, and application of chemical products. GT has been previously proposed as a suitable substance in green synthesis strategies owing to its renewable and safe nature, availability, as well as capability of acting as a reductive agent in metal nanoparticle synthesis [50,51,52]. As an illustration, Ghayempour et al. took benefits from GT as a reductant and stabilizer to prepare urchin-like ZnO nanorod arrays (diameter and length of 55–80 nm and 240 nm, respectively) at low-temperature by applying ultrasonic treatment [53]. It was demonstrated that hydroxyl and carboxyl groups of GT were oxidized. In another study, Darroudi et al. could successfully synthesize mono-dispersed nanoceria particles with a small size (20 to 40 nm) by using GT [54]. The authors introduced GT as a proper stabilizing substance for the green biosynthesis of nanoparticles, which shows comparable efficiency to conventional reduction methods using hazardous polymers or surfactants (Figure 3).

## 5. GT for Wastewater Treatment

In recent years, industrial development has led to the overproduction of industrial wastewater and environmental pollution. Materials with natural origins (e.g., plants) are commonly used for the removal of heavy metal ions and dyes from different wastewaters. The main advantages of plant-derived substances for such applications are stated as their relative safety, low cost, free supply, and relatively simple technological processing [55,56,57,58]. GT with primary and secondary hydroxyl, carboxylic acid, and epoxy groups in its structure provides a desirable platform for reactions with different reagents bearing specific functional groups [59,60]. As an eco-friendly substance, GT seems a suitable candidate for waste removal applications [61]. However, some physico-chemical properties of GT (e.g., high solubility in water) should be improved; hence, various approaches for the modification and fabrication of GT-based composites have been proposed to overcome such limitations [62,63]. For instance, Shojaipour et al. developed bioadsorbent hydrogels made of GT and trimethoxysilane quaternary ammonium (TMSQA) (as cross-linker) to remove NO_3_^−^ ions from water [64]. This system showed the capability of removing 98.26% of NO_3_^−^ ions under an optimal adsorption condition (contact time = 20 min, adsorbent dosage = 30 mg, pH = 7, and initial nitrate concentration = 30 mg/L) and the maximum monolayer adsorption capacity was recorded as 21 mg/g at 298 K. The authors stated that the adsorption process is spontaneous and exothermic (ΔGº  =  −89.1 kJ mol^−1^) in nature, which follows the pseudo-second-order rate kinetic and the obtained data are fitted with the Langmuir isotherm. It should be mentioned that the composites made of GT are also prepared for potential usage in waste removal strategies [65,66]. Recently, a new eco-friendly nanocomposite of CoFe_2_O_4_ modified with GT was successfully prepared to remove acid dyes from aqueous solutions; GT could significantly improve the adsorption properties and surface morphology of the sorbent [67].

## 6. GT for Drug Delivery Strategies

**Figure 4:**
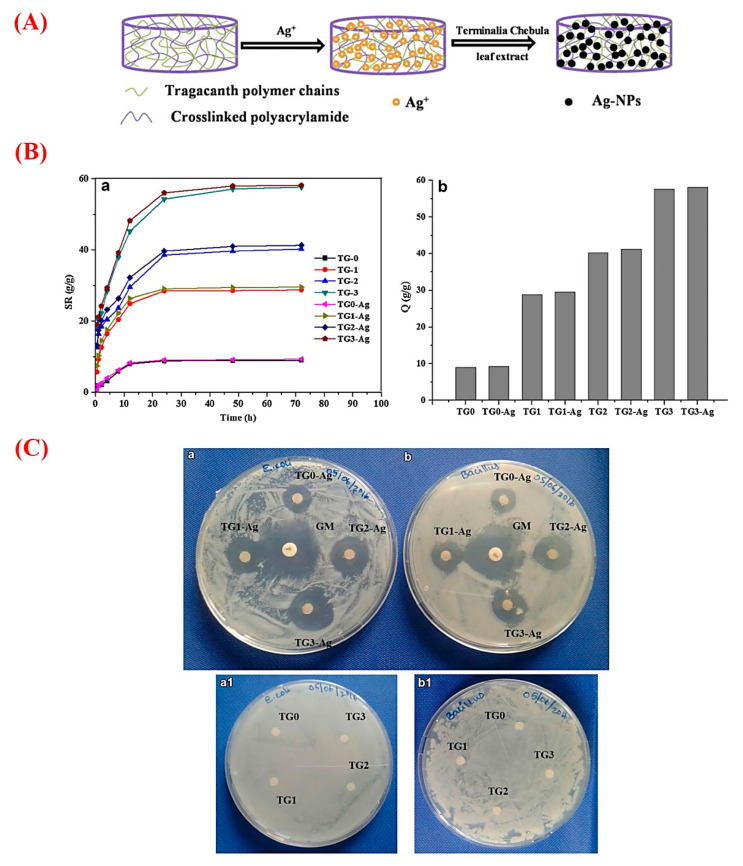
(**A**) Schematic representation of the formation of silver nanoparticles (Ag-NPs) in gum tragacanth (TG) hydrogel networks. (**B**) Graphs a and b exhibiting swelling and equilibrium swelling ratio Q (g/g) of the TG hydrogels (TG0, TG1, TG2, and TG3) and their Ag-NPs embedded counterparts (TG0-Ag, TG1-Ag, TG2-Ag, and TG3-Ag), respectively. (**C**) The results of the antibacterial activity of the Ag-NPs containing TG0, TG1 TG2, and TG3 hydrogels and the pristine TG hydrogels on (a/a1) *E. coli*, and (b/b1) *B. subtilis* [Gentamicin (GM) antibiotic is as the positive control]. Note: TG0, TG1, TG2, and TG3 contain 0, 100, 200, and 400 mg of GT. Reproduced with permission from [75], Copyright 2017, Springer Nature.

In recent years, plant-derived polymers have evoked tremendous interest in the pharmaceutical setting, such as drug delivery approaches [68]. GT possesses the necessary criteria of an appropriate drug release vehicle due to its excellent biocompatibility, biodegradability, and the potential of loading wide ranges of natural and synthetic bioactive molecules [69]. Accordingly, a variety of chemicals and drugs were loaded into different GT-based constructs (e.g., nanogels, hydrogels, and nanofibers) and relevant composites to be delivered to desirable sites via oral or other administration routes [70]. Antibacterial, anti-cancer, anti-inflammatory, and antioxidant agents are among the most delivered therapeutics by GT and its composites [49,71,72]. For antibacterial applications, GT in the form of hydrogels and nanogels has been widely used as a delivery platform for organic (e.g., plant extracts) and inorganic (silver nanoparticles) substances [73,74]. As an illustration, Rao et al. added silver nanoparticles to GT hydrogels to impart the antibacterial ability to the construct [75]. They prepared GT/acrylamide (AAm) hydrogels via the standard redox polymerization method and then synthesized silver nanoparticles (Ag-NPs) in GT hydrogels (Figure 4). The results showed an increment in the swelling ratio of the hydrogels along with increasing the amount of GT. Moreover, embedding Ag-NPs into the hydrogels caused a small increase in the swelling capacity in comparison to pure counterparts. As shown in Figure 4, the saturation of all the hydrogels happened within 3 days. The authors reported that this composite hydrogel, being able to effectively inhibit Gram-positive (*Bacillus subtilis* (*B. subtilis*)) and Gram-negative bacteria (*Escherichia coli* (*E. coli*)), could be a suitable candidate for wound healing as well as water purification applications.

For delivery of the anti-cancer drug cisplatin (CP), composite nanogels (size = 58–70 nm) of GT and lecithin (LC) were previously proposed in which the drug was embedded in the GT core and remained covered by LC as shell [76]. It has been reported that adding GT to polymeric drug delivery systems may improve their physico-mechanical properties and sustained release profile. In this regard, Apoorva et al. incorporated GT into pH-sensitive sodium alginate (SA) hydrogels and evaluated its effects on the release profile of the loaded drug, i.e., phenolic compounds extracted from *Basella* sps [77]. Indeed, they added GT to SA to overcome one of the limitations of pure alginate beads, i.e., the low entrapment efficiency. A series of SA-GT beads were prepared using an ionic gelation method in which different ratios of SA and GT (AT0 = only SA, AT11 = 1:1, AT12 = 1:2, and AT21 = 2:1) were poured into distilled water. The resulted droplets were mixed with calcium chloride hardening solution (2% *w*/*v*) to make the beads with a size ranging from 638 μm to 798 μm. The obtained results revealed higher swelling behavior of SA formulations in simulated intestinal fluid (SIF) after the incorporation of increasing GT content. This could be due to the hydrophilic nature (i.e., OH and COOH groups) of GT that interact with water molecules leading to promoted swelling behavior [43]. A significantly higher encapsulation efficiency (ranging from 62% to 78%) was also documented for the phenolic compounds in the SA-GT beads. This might be affected by large vacant space between the polymeric chains to incorporate phenolic agents inside the loop structures and subsequent hydrogen bonds formation with the OH groups of GT. In addition, the sequential and controlled release of the phenolics in the simulated intestinal environment (SIE) was recorded in the groups AT21, AT11, and AT12, leading to the high absorption (99%) of the extracts in an in vitro model of the small intestine. GT nanofibers were recently produced by using a sonochemical/microemulsion method for the controlled delivery of peppermint oil [78]. The prepared GT nanofibers, having one-dimensional shape (58 nm thickness and 1 µm length), showed the ability to allow the controlled release of peppermint oil (92.38% of the drug after 18 h), rendering antibacterial activities against *E. coli* and *S. aureus* without no significant toxicity over human fibroblast cells. The usefulness of GT nanofibers in drug delivery approaches is also documented elsewhere [79].

## 7. GT in Tissue Engineering and Regenerative Medicine

Tissue engineering and regenerative medicine (TERM) is a multidisciplinary field, which comprises molecular and cellular biology, chemistry, and materials science with the aim of regenerating a damaged tissue both structurally and functionally. The extracellular matrix (ECM) is recognized as a key player in the regenerative process of a broad range of human tissues as to its ability to provide a suitable biological substrate for improving cell adhesion, proliferation, migration, and differentiation. As the ECM is mainly composed of proteoglycans, glycosaminoglycans, glycoproteins, and glycolipids, polysaccharides have been considered as promising materials for generating biomimetic scaffolds [80,81,82,83]. Natural polysaccharides (e.g., GT and alginates) are being extensively used in TERM strategies thanks to their biocompatibility and biodegradability, structural and functional diversity, as well as their availability and renewability as compared to synthetic polymers [84]. Still, some drawbacks are mentioned with natural polysaccharides, such as their rapid degradation that can endanger the mechanical and biological properties of the scaffold. Current research has focused on these limitations and brought innovative approaches, including their reinforcement by copolymerization with other biomaterials and physicochemical crosslinking [85]. It is worth mentioning that improved physico-mechanical properties (e.g., structural stability) of polysaccharide-based scaffolds may also lead to an accelerated tissue regeneration [86].

It is possible to fabricate several tissue-engineered constructs containing GT, which could be useful in the acceleration of the tissue healing process. In the following sections, we summarize the potential applications of different GT-containing structures (e.g., nanofibers, hydrogels) in the concept of TERM.

### 7.1. In Vitro Cell Interactions

The evaluation of the biocompatibility of GT has a long history; Hagiwara et al. reported GT as a non-carcinogenic substance in 1992 [87]. Recent studies have also confirmed the compatibility of GT with living systems, including different mammalian cells. For example, Singh et al. presented GT hydrogels as non-thrombogenic and haemo-compatible materials with the ability to deliver the anti-cancer drug methotrexate in a controlled and sustained manner [88]. In another study, Fattahi et al. investigated the cytotoxicity of the soluble modified fraction of A. gossipinuson-derived GT on Hela and HepG2 cancer cell lines as well as L929 fibroblast cell line. Their obtained results showed no adverse effects of GT on two cancer cells, while a slight improvement in cell viability was observed for the L929 cell line [47]. In a recently published study, bacterial cellulose/keratin electrospun nanofibers were reinforced by GT for generating a suitable substrate for mammalian cell culture. The experimental data showed an improvement in tensile properties and wettability of the polymeric fibers, as well as better attachment and proliferation of L929 fibroblast cells onto the modified scaffolds [89]. It is worth noting that the anionic nature of GT may play a critical role in its biocompatibility and targeted delivery of therapeutic agents, as it has been shown by synthetic anionic polymers [90,91,92].

### 7.2. Hard Tissue Regeneration

**Figure 5:**
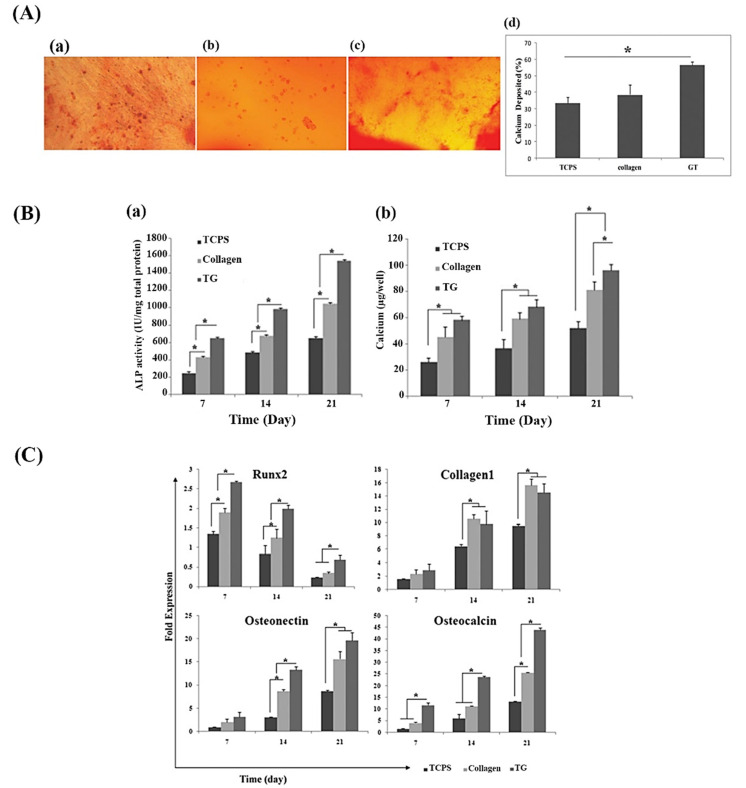
(**A**) Microscopic photographs of differentiated human adipose-derived mesenchymal stem cells cultured on tissue culture polystyrene (TCPS) (**a**), collagen (**b**), and gum tragacanth (GT) (**c**) stained with Alizarin Red S after 21 days of culturing in the osteogenic induction medium (Magnification × 40), as well as the graph (**d**) showing the quantified values for calcium deposition of different groups. (**B**) Alkaline phosphatase (ALP) activity of (**a**) and calcium content (**b**) of the stem cells cultured on TCPS, collagen, and GT after 7, 14, and 21 days of culturing in the osteogenic differentiation medium (asterisks significant difference between the groups at *p* < 0.05). (**C**) Real-time PCR data exhibiting relative expression of osteogenic-related genes including Runx2, collagen type 1, osteonectin, and osteocalcin in the stem cells cultured on TCPS, collagen, and GT at day 7, 14, and 21 (* refers to the significant difference between the groups at *p* < 0.05). Reproduced with permission from [16], Copyright 2016, Elsevier.

Bone tissue plays a critical role in the human body from both structural and functional aspects. The high rate of bone injuries and damages resulted from traumas, cancers, and genetic abnormalities is a big challenge in the clinic and demand tissue substitutes. Up to now, huge numbers of cells, materials, and bioactive molecules have been used to prepare suitable constructs for the replacement of injured bones [93,94,95,96]. In the case of bioactive molecules, several studies have shown the effectiveness of a variety of naturally-derived substances (e.g., curcumin) [97,98]. On this object, the use of GT in BTE applications has gradually grown; however, more research is needed to reveal details about the cellular and molecular mechanisms regulated by GT in the bone regeneration process [99,100]. In a pioneering study, Kulanthaivel et al. prepared GT calcium alginate beads as a cell encapsulation system and evaluated their proangiogenic and osteogenic properties [17]. The GT-incorporated beads were produced by the ionic gelation method in which GT was added to the alginate solution in a concentration of 0, 25, 35, and 50 (*w*/*v*). They reported that the incorporation of GT in the calcium alginate bead yielded an improvement in transport, swelling, and degradation properties. Moreover, cell experiments revealed improved viability, growth, and differentiation of bone cells (MG-63 cells) encapsulated in the GT-containing samples as compared to GT-free control. As a reliable marker of angiogenesis, the expression of the HIF-1α was up-regulated to 1.45, 1.40, and 1.23 folds in GT25, GT35, and GT50 samples as compared to the GT0, respectively. In another study, Haeri et al. evaluated the osteogenic potential of GT (25 mg/mL)-containing collagen hydrogels on human adipose-derived mesenchymal stem cells (h-ASCs) [16]. In vitro assessments showed that GT-containing samples had no cytotoxic effect and could improve alkaline phosphatase (ALP) activity as well as mineralization in the cells in comparison to controls (see Figure 5). Based on this evidence, the authors claimed that GT-containing hydrogels could be a useful scaffold for orthopedic applications.

Recently, GT has also been used as a natural binder for the fabrication of hydroxyapatite (HAp) scaffolds by using a polymer replication method [101]. The binding ability of GT and its effects on the mechanical properties and porosity of HAp scaffolds were evaluated. The obtained data demonstrated the possibility of fabricating scaffolds with highly interconnected macropores along with smaller micropores (400–600 µm and 2–10 µm, respectively) and appropriate compressive strength (0.036 MPa to 2.954 MPa), which are favorable for non-load-bearing applications. Also, in vitro studies using Vero cells demonstrated cytocompatibility of the samples during culturing for 24 h.

Furthermore, poly(lactic-co-glycolic acid) (PLGA)/GT core-shell electrospun nanofibers have been proposed for periodontal regeneration as they could serve as a suitable platform for antibiotic loading and delivery [102].

### 7.3. Soft Tissue Healing

Apart from being proposed for hard tissue regeneration, GT has recently attracted much attention in the repair and restoration of soft tissues, including skin and nerve [103,104]. It has been shown that topical administration of GT could accelerate the closure of full-thickness skin wounds in rats [105]. Therefore, several attempts have been made to take benefit of GT in this sense; for example, Zarekhalili et al. suggested the use of poly(vinyl alcohol) (PVA)/GT/polycaprolactone (PCL) hybrid nanofibrous scaffolds as suitable skin substitutes [106]. They showed that the introduction of PVA and PCL in the formulation not only facilitated the electrospinning process of the GT solution but also improved the mechanical properties of the electrospun nanofibers. Moreover, the prepared scaffolds could support the growth and proliferation of NIH 3T3 fibroblast cells.

**Figure 6:**
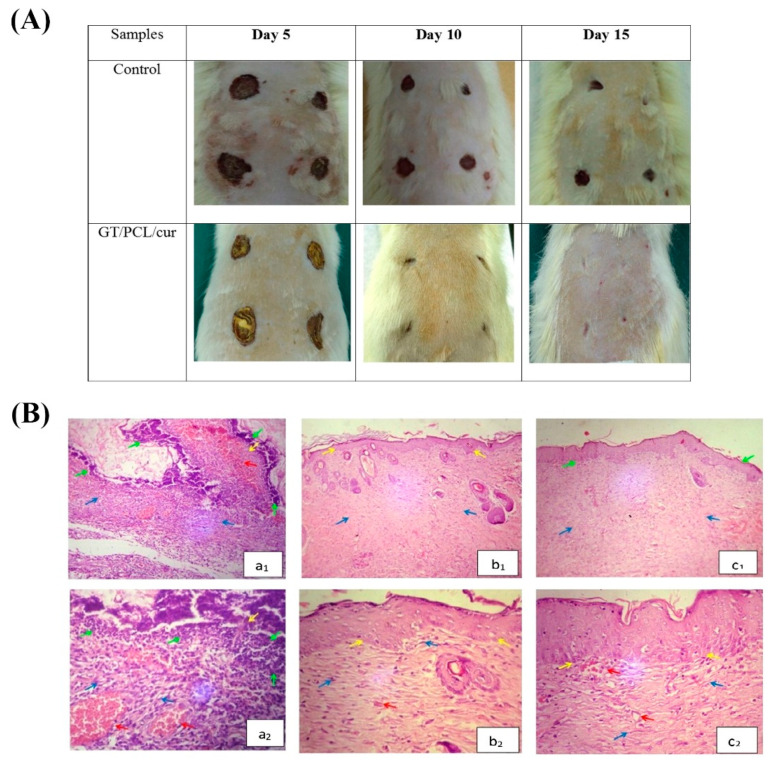
(**A**) Macroscopic observation of the wound closure in un-treated diabetic rats and the animals treated with gum tragacanth (GT)/poly vinyl alcohol (PVA) electrospun nanofibers containing 3% curcumin (Cur) at 5, 10, 15 days post-surgery. (**B**) Microscopic observations of H&E stained slides of the untreated skin wounds (**a1**,**a2**) and those treated with the PCL/GT/Cur nanofibers (**b1**,**b2**) and the PCL/GT/Cur loaded with umbilical cord Wharton jelly-derived mesenchymal stem cells (**c1**,**c2**) after 15 days of surgery. Note that granulation tissue, epithelial regeneration, angiogenesis, and collagen fibers were indicated by blue, yellow, red, and green arrows, respectively. Magnification of a1, b1, c1 is 100×, and a2, b2, c2 is 400×. Reproduced with permission from [108], Copyright 2016, Elsevier.

Since GT is recognized as an appropriate drug delivery system, a variety of natural and synthetic substances have been loaded into scaffolds made of GT combined with other polymers. In this regard, Ranjbar-Mohammadi and Bahrami presented PCL/GT nanofibers as promising vehicles for the efficient and sustained delivery of curcumin (Cur), which could improve fibroblast cell growth in vitro and may have significant therapeutic potential as a wound dressing [107]. These electrospun nanofibrous scaffolds (2:1 PCL/GT mass ratio) containing 3% Cur were further implanted in diabetic rats to evaluate their skin wound healing capacity in vivo; the results confirmed both their biocompatibility and regenerative potential (Figure 6) [108].

In another study, GT was used as a novel “green-wound-healing” product for encapsulation and delivery of Aloe Vera extract [53]. As inhibiting bacterial infections and reducing the pain are of great importance in wound injuries, GT/PVA/PVP-based hydrogels were loaded with gentamicin and lidocaine as antibiotic and analgesic drugs, respectively [14]. Based on the reported results, the hydrogels showed the capability of wound fluid absorption and slow drug release. In addition to blood compatibility, the samples showed an excellent permeability to water vapor and O_2_ while were impermeable to microorganisms.

Some researchers have also proposed the application and usability of GT in peripheral nerve regeneration strategies. In 2016, Ranjbar-Mohammadi et al. fabricated GT/poly(L-lactic acid) (PLLA) electrospun nanofibrous scaffolds. For this purpose, they mixed various ratios (*w*/*w*) of GT and PLLA as 0:100, 25:75, and 50:50 to prepare aligned and random constructs [104]. The cell experiments showed that aligned GT/PLLA 25:75 was the best composition for nerve cells (PC12 cell line) growth and supported the expression of bi-polar neurite extensions and the orientation of the cells.

## 8. Concluding Remarks and Future Perspectives

GT is known as a versatile natural substance derived from different species of the genus Astragalus. GT has a long successful history in food and pharmaceutical formulation; furthermore, it has been gradually found to be a useful material in other areas of biomedicine, including waste management, green synthesis of nanoparticles, drug delivery strategies, and tissue engineering and regenerative medicine [11]. The main reasons for the extended usage of GT could be summarized as its biocompatibility and ease of chemical modifications [88,109]. However, the high cost and availability of xanthan gum (mostly found in Iran and Turkey) as a cheaper competitor limit the demand of GT as regards use in pharmaceutical and industrial settings [6,110]. Recently, GT has attracted much interest in tissue engineering strategies addressed to both hard (e.g., the bone) and soft (e.g., the skin) tissues. Although not many studies on GT-based therapies for tissue reconstruction have been reported so far, there is convincing evidence that supports the suitability of GT-based constructs (e.g., hydrogels and nanofibers) for accelerating the wound healing process. In this sense, the capability of GT in the loading and delivery of bioactive molecules, as well as the possibility of easily making composites, may be considered as promising points for boosting the regeneration of damaged tissues [111]. Another important issue deserving investigation concerns the critical comparison between GT and other polysaccharides used in tissue engineering and regenerative medicine, in order to elucidate whether GT, besides being a valuable alternative, is truly superior to the other existing options. Such a comparison should involve not only direct biological effects but also indirect effects like those mediated by physical and mechanical properties of GT. It has been shown that, for example, biomaterial elasticity can guide stem cell differentiation [112] and cell activity can be affected by stiffness gradients of the substrate [113]. The understanding of all the aspects through which cells can “sense” biomaterials, which in turn influence cell metabolism, is the key to developing new and truly functional tissue-engineering approaches.
